# Surface Electrical Impedance Myography Detects Skeletal Muscle Atrophy in Aged Wildtype Zebrafish and Aged *gpr27* Knockout Zebrafish

**DOI:** 10.3390/biomedicines11071938

**Published:** 2023-07-07

**Authors:** Seward B. Rutkove, Zsu-Zsu Chen, Sarbesh Pandeya, Santiago Callegari, Tyler Mourey, Janice A. Nagy, Anjali K. Nath

**Affiliations:** 1 Department of Neurology, Beth Israel Deaconess Medical Center, Boston, MA 02215, USA; srutkove@bidmc.harvard.edu (S.B.R.); jnagy@bidmc.harvard.edu (J.A.N.); 2 Department of Endocrinology, Beth Israel Deaconess Medical Center, Boston, MA 02215, USA; 3 Department of Cardiology, Beth Israel Deaconess Medical Center, Boston, MA 02215, USA; 4 Zebrafish Core Facility, Beth Israel Deaconess Medical Center, Boston, MA 02215, USA; 5 Broad Institute, Cambridge, MA 02142, USA; 6 Department of Medicine, Harvard Medical School, Boston, MA 02115, USA

**Keywords:** aging, sarcopenia, zebrafish, skeletal muscle, bioelectrical impedance, electrical impedance myography, g-coupled protein receptors, GPR27, genetic screening, therapeutic targets

## Abstract

Throughout a vertebrate organism’s lifespan, skeletal muscle mass and function progressively decline. This age-related condition is termed sarcopenia. In humans, sarcopenia is associated with risk of falling, cardiovascular disease, and all-cause mortality. As the world population ages, projected to reach 2 billion older adults worldwide in 2050, the economic burden on the healthcare system is also projected to increase considerably. Currently, there are no pharmacological treatments for sarcopenia, and given the long-term nature of aging studies, high-throughput chemical screens are impractical in mammalian models. Zebrafish is a promising, up-and-coming vertebrate model in the field of sarcopenia that could fill this gap. Here, we developed a surface electrical impedance myography (sEIM) platform to assess skeletal muscle health, quantitatively and noninvasively, in adult zebrafish (young, aged, and genetic mutant animals). In aged zebrafish (~85% lifespan) as compared to young zebrafish (~20% lifespan), sEIM parameters (2 kHz phase angle, 2 kHz reactance, and 2 kHz resistance) robustly detected muscle atrophy (*p* < 0.000001, q = 0.000002; *p* = 0.000004, q = 0.000006; *p* = 0.000867, q = 0.000683, respectively). Moreover, these same measurements exhibited strong correlations with an established morphometric parameter of muscle atrophy (myofiber cross-sectional area), as determined by histological-based morphometric analysis (r = 0.831, *p* = 2 × 10^−12^; r = 0.6959, *p* = 2 × 10^−8^; and r = 0.7220; *p* = 4 × 10^−9^, respectively). Finally, the genetic deletion of *gpr27*, an orphan G-protein coupled receptor (GPCR), exacerbated the atrophy of skeletal muscle in aged animals, as evidenced by both sEIM and histology. In conclusion, the data here show that surface EIM techniques can effectively discriminate between healthy young and sarcopenic aged muscle as well as the advanced atrophied muscle in the *gpr27* KO animals. Moreover, these studies show how EIM values correlate with cell size across the animals, making it potentially possible to utilize sEIM as a “virtual biopsy” in zebrafish to noninvasively assess myofiber atrophy, a valuable measure for muscle and gerontology research.

## 1. Introduction

Humans exhibit a progressive loss of skeletal muscle mass and function, which typically begins at age 30 and accelerates with advanced age into a condition called sarcopenia [1,2,3]. The most common correlate of muscle atrophy is age [4]. Sarcopenia is associated with a significant decrease in motor performance, including reduced maximal strength and slower contractile velocity, in addition to increased variability in motor performance when repeating a motor task [5,6]. Approximately 45% of adults over 60 experience a decline in motor performance that causes moderate to severe physical limitations which impacts their ability to perform the routine activities of daily life [7]. Sarcopenia is a major risk factor for increased frailty, falls, hospital admissions, and death [8,9,10,11]. Muscle loss, analogous to sarcopenia, may play an etiological factor in many neuromuscular disorders, including amyotrophic lateral sclerosis, muscular dystrophies, and critical illness myopathy [3,12,13,14]. The healthcare burden of skeletal muscle deficits is increasing due to a higher proportion of older adults, projected to reach 2 billion people worldwide in 2050 [15]. Currently, there are no pharmacological treatments for sarcopenia [3]. In addition, there exist few whole-animal vertebrate model systems in which it is practical to conduct high-throughput chemical and genetic screens for the discovery of novel small molecules and therapeutic targets for age-related sarcopenia [16]. Zebrafish is a promising vertebrate model that could fill this gap and accelerate the pace of drug discovery in the field of age-related sarcopenia [16].

Concordant with other vertebrate models of aging, zebrafish exhibit gradual senescence throughout their lifespan [17,18,19]. Although zebrafish live slightly longer than mice, zebrafish possess unique features that are advantageous to gerontology research [20]. The zebrafish genome and disease-causing genes are highly conserved with humans [21,22]. There is also high conservation in the molecular pathways regulating skeletal muscle biology between these two species, in addition to the fundamental mechanisms of aging [20,23,24,25,26,27,28]. Moreover, the zebrafish model has attributes that make it a robust and unique model for studying aging. Zebrafish is a vertebrate model that produces hundreds of offspring every week, and adult animals can be housed more densely and far less expensively than mice, which is important given the long-term nature of aging studies. In addition, dozens of transgenic lines are routinely carried within each zebrafish laboratory, and fluorescent reporter lines—generated in transparent wildtype zebrafish—can be leveraged to conduct imaging of organs and cell types in living adult animals. Thus, zebrafish have several advantages as a preclinical model for sarcopenia.

Current methods to evaluate zebrafish skeletal muscle health in aging adult animals include histology, swimming performance, and molecular markers in tissue lysates. These methods have important limitations as endpoints in zebrafish studies aimed at investigating the etiology of and candidate treatments for sarcopenia. For example, histological analysis of the trunk musculature requires euthanasia, and while swimming performance is non-invasive, this measure reflects both muscle function in addition to animal behavior. In this study, our objective was to develop a non-invasive, rapid, and quantitative method to assess skeletal muscle health in adult zebrafish. To develop this technology, we modified an established technology, termed surface electrical impedance myography (sEIM), which has been used to quantitatively evaluate skeletal muscle in healthy young animals versus aged animals, including mice, rats, dogs, and humans [29,30,31,32,33], in addition to animal and human muscular dystrophies [33,34,35,36].

In the sEIM procedure, a standard four-electrode array is placed on the skin’s surface. The electrode measures the voltage following the application of a high-frequency, low-amplitude current that is forced through a small region of tissue; the resulting spectral data are plotted. Compared to other muscle assessment techniques used in the clinic, i.e., electromyography, nerve conduction studies, and magnetic resonance imaging, sEIM does not require expensive equipment, extensive training, or software-based image analysis and, importantly, can easily be implemented in both primary care settings and experimental settings; moreover, it is quantitative, thus allowing it to serve as a valuable biomarker [34,37,38]. In addition, as compared to a muscle needle biopsy procedure, which is invasive and requires histology processing in combination with labor-intensive software-assisted morphometric analysis, sEIM data acquisition is painless, and sEIM data analysis is significantly faster.

In sEIM, the measured impedance values are affected by the compositional and structural features of skeletal muscle and its biophysical properties (i.e., resistive and capacitive properties). For example, the in vivo features detected with this technology include myocyte size, shape, and membrane properties, in addition to the amount of endomysium (connective tissue) and intra-cellular and extra-cellular matrices (i.e., fat and connective tissue). The impedance data generated are termed reactance (generated by the capacitive elements present in tissues, i.e., cell membranes, connective tissue, fat, etc.), resistance (generated by flow through extracellular and intracellular ionic fluids), and phase angle (a geometric relationship between reactance and resistance). Together, these bioelectrical values generate a “virtual biopsy” of the skeletal muscle.

There are several notable features of sEIM that are particularly advantageous for assessing skeletal muscle health in adult zebrafish. First, the sEIM procedure is fast: data are collected in <1 min and graphed in real-time. In comparison, several weeks are required to complete the steps of fixation with decalcification of zebrafish bones, histological processing of samples, and quantitative image-based morphometric analysis of muscle tissue sections. sEIM is also faster and less labor-intensive than fluorescent-based confocal microscopic analyses of adult animals. Second, the procedure for sEIM is painless and noninvasive in zebrafish, allowing repeated measures to be obtained in the same animal. Third, sEIM generates reproducible and quantitative data points (phase angle, reactance, and resistance values), which will provide robust metrics for adult zebrafish chemical screens of muscle phenotypes. Therefore, establishing surface electrical impedance myography in zebrafish—an exceptionally tractable model organism—will open new and exciting paths of investigation into sarcopenia.

Here, we developed a platform to noninvasively collect bioimpedance measurements from the surface of zebrafish trunk musculature, and then evaluated if these measurements were sensitive biomarkers of skeletal muscle health in adult zebrafish while also comparing them to needle-based measurements (an invasive and terminal procedure in zebrafish). We tested the hypothesis that surface electrical impedance myography detects muscle atrophy in aged zebrafish adults, in addition to the effects of genetic perturbations that affect zebrafish skeletal muscle health during aging. In aged zebrafish as compared to young zebrafish, sEIM parameters (phase angle, reactance, and resistance) robustly detected muscle atrophy. Moreover, sEIM values exhibited strong correlations with an established morphometric parameter of muscle atrophy (myofiber cross-sectional area), as determined by histological-based morphometric analysis. In addition, we determined the impact of *gpr27,* an orphan GPCR and druggable target, on skeletal muscle health in zebrafish. We chose this specific gene because it is part of an ongoing project aimed at assigning biological functions to understudied, druggable genes; in that study, we detected defects in skeletal muscle biology in *gpr27* KOs as compared to sibling WT controls. Here, using sEIM, myofiber atrophy was detected in *gpr27* KOs, consistent with the histology findings; moreover, exacerbated skeletal muscle atrophy was detected in aged *gpr27* KOs as compared to aged WT siblings. In sum, we demonstrate that sEIM-based assessment of zebrafish skeletal muscle health enables a new, fast, non-invasive approach to investigate skeletal muscle atrophy in aged, sarcopenic zebrafish.

## 2. Materials and Methods

Zebrafish. All studies were performed using approved animal protocols from the BIDMC Institutional Animal Care and Use Committee (IACUC). In addition, all studies were performed in accordance with the National Institute of Health’s Guide for the Care and Use of Laboratory Animals. Animals were housed in mixed-sex tanks on a recirculating system and maintained at 28.5 °C on a 14/10 h light/dark cycle. In this study, Tübingen-strain zebrafish were used. Young animals were 8–12 months of age, and aged animals were 36 months of age. Note that the literature reported that wildtype zebrafish lifespan is approximately 3.5 years [39]. In addition, fecundity has drastically declined by 2 years of age. This is consistent with the lifespan and fecundity in our Tübingen colony. Therefore, we chose 3 years of age (~85% of their lifespan) to classify animals as “aged”. Given that zebrafish become sexually mature adults at the age of 3 months, and in our zebrafish facility, the maximal success rate of breeding occurs between 6 and 18 months of age, we “retire” wildtype breeding lines at 18 months of age due to decreased fecundity. Therefore, in this study, we classified “young” animals as 8–12 months of age or ~20–30% of their lifespan. *gpr27* knockout zebrafish and sibling WT controls were generated as previously described [40]. Animals were derived from heterozygous incrosses. In all studies, both males and females were used in approximately equal numbers.

Morphometric measurements. Prior to the start of each assay, a picture was taken of each animal in order to measure the “standard length” of the animal’s body, which excludes the length of the caudal fin. Body weight was also measured after removing excess water via placement on a paper towel.

**Figure 1:**
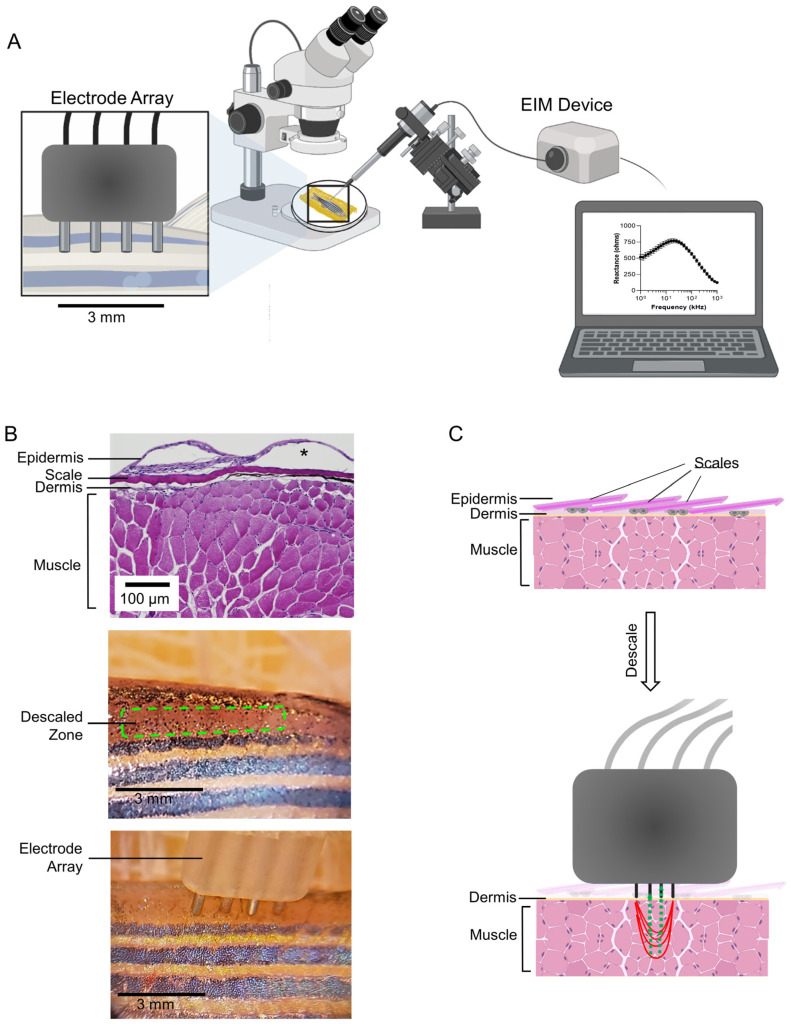
Surface electrical impedance myography in zebrafish skeletal muscle: Experimental set-up. (**A**) Cartoon depiction of the experimental procedure. The measurements were completed in <1 min and graphed in real-time. (**B**) H&E-stained tissue section of a region of epaxial caudal muscle (note the asterisk denotes an artifact of histology; in living zebrafish, the epidermis is in contact with and covers the scale). Image of descaled caudal musculature. The descaled region is outlined with a green dashed line. Image of the 4-pin surface electrode placed on top of the descaled caudal musculature, and positioned along the anterior–posterior axis below the dorsal fin. (**C**) Cartoon depiction of the surface electrode during data acquisition and tissue layers at the site of measurement (note this drawing is not to scale). The red lines depict the low-amplitude, high-frequency alternating electrical current that is applied to the muscle through a pair of outer electrodes, while the green lines represent the resulting voltages that are measured through a second pair of inner electrodes (created in BioRender.com).

Surface electrical impedance myography measurements. The EIM surface electrode was constructed by inserting four 27-gauge subdermal Neuroline^TM^ stainless-steel needle electrodes (Ambu^®^, Columbia, MD, USA) into a custom-made resin holder (Form 2 desktop 3D printer, Formlabs, Somerville, MA, USA), designed such that the electrodes are spaced 1 mm apart [41]. The needle electrodes were secured in place in the resin holder using UV-activated clear glue (Greatfishing Fly Tying UV Clear Glue, Amazon, Bellevue, WA, USA), followed by heat shrink tubing (Koowin Heat Shrink Tubing Kit, Amazon). Following curing, the tips of the four needle electrodes were blunted by repeated passage of the electrode tips over a graded series of sandpaper (Miady 120-3000 Assorted Grit, Amazon). The resultant blunt tips of the four electrodes each protrude 1 mm from the end of the holder. The other ends of the electrodes were stripped off, and their connectors were soldered to the wires that connect directly to the mView impedance-measuring device (Catalogue# EIM1103, Myolex^®^, Inc, Boston, MA, USA). The mView device is connected to a laptop and controlled by mView software (Myolex^®^, Inc, Boston, MA, USA). The surface electrode was affixed to a micromanipulator (Catalogue# M3301, World Precision Instruments, Sarasota, FL, USA), and its position and angle were adjusted under a stereomicroscope (Figure 1A).

The day before the sEIM study, zebrafish were anesthetized in Tricaine S. The zebrafish was placed on moist gauze (soaked in Tricaine S) under a stereomicroscope. Another moist gauze was placed on top of the zebrafish to keep it moist and in place during the procedure. A watchmaker’s tweezer (with blunted tips, which were sanded with a fine metal file) was used to gently lift upwards and then pluck outwards 8–12 scales from the middle of the dorsal fin up in a rostral direction until an area of approximately 6 mm × 3 mm was descaled. The next day, sEIM measurements were taken. Animals were anesthetized, and then excess water was removed by briefly placing the animal on a paper towel. The zebrafish were placed on a wedge platform such that the animal would lie at an angle of 15°. The approach angle of the surface electrode (attached to a micromanipulator) was set at 50° (Figure S1A). The electrode was positioned on descaled, epaxial caudal musculature just below the dorsal fin—a morphological landmark that was used to consistently insert the electrode in the same region on all animals (Figure 1A,B). The probe was placed in the descaled zone such that the blunt ends of the four electrodes were firmly in contact with the skin, but the pressure was not enough to sink the skin (Figure S1B). A low-amplitude, high-frequency alternating electrical current (400 µA, 41 frequencies between 1 kHz and 1 MHz) was then applied to the muscle through the pair of outer electrodes, and the resulting voltages were measured via a second pair of inner electrodes (Figure 1C). The procedure was completed in <1 min, during which 4 serial measurements were acquired. Subsequently, the animal was returned to their home tank.

Needle electrical impedance myography measurements. The EIM needle electrode was made as previously described [42], and their connectors were soldered to the wires that connect directly to the mView impedance-measuring device (Catalogue# EIM1103, Myolex^®^, Inc., Boston, MA, USA). Similar to the surface electrode, in the needle electrode the outer pair of electrodes delivered alternating electrical currents while the inner pair measured the resulting voltage. Due to the invasive nature of needle EIM in the small zebrafish, zebrafish were first euthanized in Tricaine S 500 mg/L. The needle electrode was designed to pierce a depth of 1 mm. The needle electrode punctured through the elasmoid scales and was inserted into the epaxial caudal skeletal muscle below the dorsal fin along the anteroposterior axis (i.e., the same spot that the surface electrode was placed) and placed dorsal to major vessels in order to prevent insertion into the dorsal aorta. Just as with the surface electrode, 4 serial measurements were rapidly captured.

Histological analysis of skeletal muscle. Dietrich’s fixative was used to fix zebrafish samples. Samples were then paraffin-embedded and sectioned cross-sectionally through the trunk skeletal musculature. Images of hematoxylin-and-eosin-stained tissue sections were captured on a Keyence BZ-X710 Imaging Platform (10×, 20×, and 60× magnification). The location of the acquired images was posterior to the placement of the electrode in order to avoid artifactual effects of injury due to the electrode or due to descaling. To measure the cross-sectional area of myofibers, Keyence BZ-X Analyzer software was used. Specifically, the hybrid cell counting tool was used to automate segmentation. However, this was manually curated for accuracy, and any segmented features that were incorrectly segmented were subsequently manually adjusted (fine edit tool). The number of myofibers analyzed per animal was ~200.

Data analysis and statistics. Data are presented as mean ± standard error of the mean. Significance was determined using Mann–Whitney tests. To correct for multiple hypothesis testing in the impedance data, we set the false discovery rate (FDR) at q < 0.05 and used a two-stage step-up method (Benjamini, Krieger, and Yekutieli; GraphPad Prism 9). In order to assess the reproducibility of EIM measurements, we calculated the mean percent difference. The following equation was used:

$$\frac{\left|V_{1}-V_{2}\right|}{\left[\begin{array}{c} \frac{\left[\begin{array}{c} V_{1}+V_{2} \end{array}\right]}{2} \end{array}\right]}\times 100$$

$V_{1}$ = Measurement 1;

$V_{2}$ = Measurement 2.

In the first analysis of reproducibility, the electrode was positioned, and 4 serial measurements were acquired. Note the electrode was not moved between the 4 measurements. We then used measurement 1 and measurement 2 to calculate the mean percent difference in the above equation. In a second analysis of reproducibility, data were collected by 2 observers on the same animal. Observer 1 captured 4 serial measurements. These were averaged to generate measurement 1 (V_1_). After observer 1 retracted the electrode, the animal and the electrode were repositioned by observer 2. Observer 2 acquired 4 measurements, whose average was used as measurement 2 (V_2_). In order to understand the relation between the measurements of the two approaches (needle and surface), we used the Spearman correlation coefficient (Rs) across the frequencies of each of the approaches. We then used this generated series of correlation coefficients in a matrix to generate heatmaps and to interpret the levels of correlations between the measurements in the resistance, reactance, and phase values. To reduce the dimensionality of the EIM data and identify the set of EIM parameters that accounts for the greatest variance in the EIM dataset, principal component analysis (PCA) was conducted using the tidymodels package in R 4.0.2 (Vienna, Austria). Animals that had missing data values were excluded. To determine if a subset of EIM variables could be used to predict age, we used a least absolute shrinkage and selection operator (LASSO) logistical regression model. Subject data were randomly assigned to the training set (80%) or the testing set (20%). The following R packages were used: glmnet and ROCR. An area under the curve (AUC) > 0.7 was used to define a good classifier model.

## 3. Results

Development of a method for surface electrical impedance myography in zebrafish adults. In our method, a micromanipulator controlled the angle and position of the electrode (Figure 1A). Attempting to acquire skeletal muscle bioelectrical impedance data by placing the electrode array directly on top of the elasmoid scales that cover the skeletal musculature was not effective. The biophysical properties of the imbricated, bony scales, namely, the resistance and capacitance, likely interfered with surface EIM measurements. Zebrafish scales are routinely lost by mechanical injuries and are replaced by new scales which regenerate within 14 days [43]. Therefore, with IACUC approval, we plucked ~8–12 scales that cover the epaxial caudal musculature below the dorsal fin (Figure 1B). Notably, the epidermis encases each scale (Figure 1B). Thus, removing a region of scales also removes the epidermis in that region. Finally, we determined that placing the animal on a wedge platform (15°) and setting the angle of the micromanipulator at 50° was optimal for ensuring the contact of the four-pin electrode array considering the curvature of the caudal musculature in zebrafish (Figure 1C and Figure S1A,B).

Both within a subject and between independent observers, our sEIM protocol was reproducible. To assess reproducibility, the surface electrode was positioned, and four serial measurements were captured while keeping the electrode in the same position on the muscle. Measurements 1 and 2 were averaged and measurements 3 and 4 were averaged. The mean percent difference was calculated between these values. In serial measurements, the mean percent difference for 2 kHz phase, 2 kHz reactance, and 2 kHz resistance was 1.47 ± 0.39, 1.92 ± 0.51, and 1.32 ± 0.35%, respectively. We observed a 1–3% variability in impedance measurements at other frequencies (Table 1; *n* = 14, 8 months of age). We also determined the reproducibility of sEIM data measurements between repeated measures in the same animal conducted by two different observers. The surface electrode was positioned on the animal by observer 1, and four serial measurements were captured. Next, the electrode was removed by observer 1. Observer 2 positioned the electrode and captured four additional serial measurements in the animal. The inter-observer measurements recorded by observers 1 and 2 were highly concordant (Table S1 and Figure S2, *n* = 14), though with a higher variability than the intra-observer measurements (Table 1). Phase, the preferred impedance parameter in many EIM studies, exhibited the highest reproducibility. Across parameters, a low frequency (i.e., 2 kHz) was more reproducible than higher frequencies (i.e., 250 kHz). Finally, we captured temporal bioelectrical impedance data by acquiring sEIM measurements on the same cohort of animals on two separate days. In this study, sEIM measurements in zebrafish skeletal muscle were acquired on day 0 and on day 14. Within this timeframe, skeletal muscle health was not expected to change in healthy animals. Concordantly, this was reflected by the impedance findings. All measured sEIM parameters (phase, reactance, and resistance) were not significantly different between the two time points (Figure S3A–L, *n* = 14).

**Figure 2:**
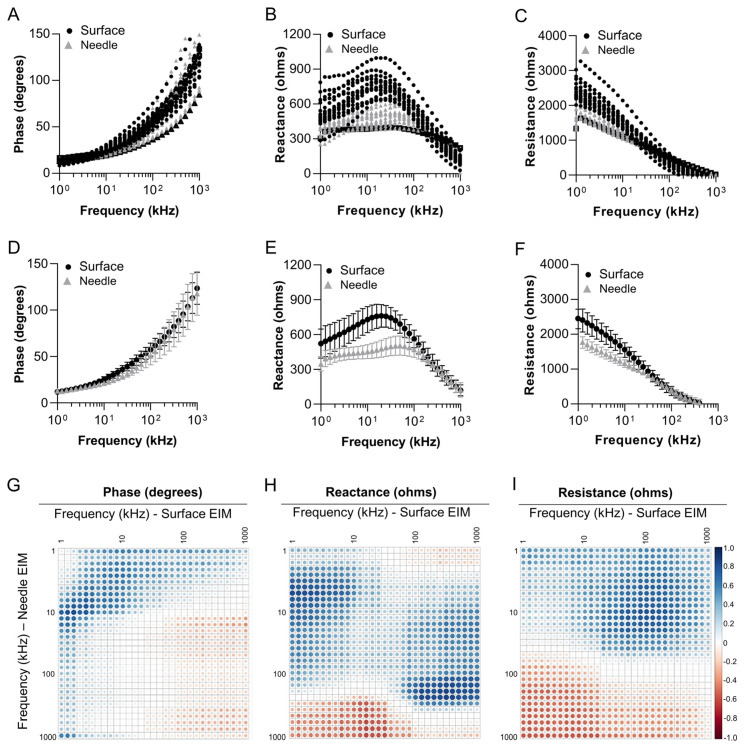
Relationships between surface EIM and needle EIM in adult zebrafish skeletal muscle. Multifrequency (1 kHz–1 MHz) graphs for EIM parameters of (**A**) phase, (**B**) reactance, and (**C**) resistance. Data displayed were acquired using a surface electrode (black) and a needle electrode (gray). Surface EIM data were collected. Then, needle EIM was collected on the same animal (*n* = 14; 8 months of age). Surface EIM and needle EIM data for each animal were plotted. Multifrequency graphs for (**D**) phase, (**E**) reactance, and (**F**) resistance are also shown as mean ± standard deviation. Correlation matrices for (**G**) phase, (**H**) reactance, and (**I**) resistance. Blue circles are positive correlations and red circles are negative correlations. White indicates zero correlation. Darker and larger circles represent higher correlation values, and lighter and smaller circles represent lower correlations.

Surface electrical impedance myography measurements correlate with needle electrical impedance myography measurements in zebrafish skeletal muscle. We next sought to compare surface muscle EIM measurements to needle-punctured muscle EIM measurements. Needle EIM methodology allows the electrode to be in direct contact with the tissues of interest; however, the needle electrode method is an invasive procedure in zebrafish given the small size of the animal in relation to the size of the needles [42]. In contrast, in the surface EIM method the electrode is placed on top of the tissue of interest (Figure 1C and Figure S1B). For this study, young animals weighing 337.6 ± 35.6 mg were used (8 months of age). A surface measurement was acquired, and immediately after, a needle measurement was acquired (Figure 2A–F). Multifrequency (1 kHz–1 MHz) phase graphs for surface and needle EIMs exhibited a similar overall shape across frequencies (Figure 2A,D). In contrast, multifrequency reactance and resistance graphs exhibited some interesting differences. The expected peak-shaped curve for reactance across frequencies was observed in the surface EIM method but not the needle EIM method (Figure 2B,E). Additionally, the expected S-shape curve for resistance across frequencies was observed with surface EIM but not with needle EIM (Figure 2C,F). In sum, in these young animals, surface EIM in zebrafish generates the overall expected trends in multifrequency impedance curves that have been previously shown in other organisms and in ex vivo tissue biopsies better than needle EIM assessments.

In order to understand the relation between the measurements of the two approaches (needle and surface), we used the Spearman correlation coefficient (Rs) across the frequencies (1–1000 kHz) for the two approaches. We generated multi-frequency correlation matrices for needle versus surface phase, reactance, and resistance values across the frequency spectrum (1–1000 kHz). In Figure 2G–I, the abscissa (x-axis) represents the frequencies from surface measurements, while the ordinate (y-axis) represents the needle frequencies. Larger spheres represent higher correlation regardless of direction, and the blue color represents positive correlation while the red color represents negative correlation. The pattern shows that lower-frequency needle measurements correlate positively to mid- to higher-frequency surface measurements, especially in resistance and reactance. It should be noted that surface electrodes can be impacted by subcutaneous fat; however, in zebrafish, the subcutaneous fat that overlays the skeletal muscle is minimal as compared to mammals (Figure 1B).

**Figure 3:**
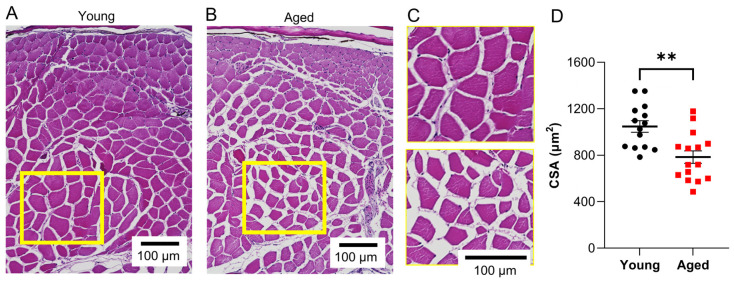
Age-related atrophy of skeletal muscle fibers in wildtype zebrafish. Representative H&E-stained images depicting epaxial caudal musculature in (**A**) young (8 months (~20% of lifespan)) and (**B**) aged zebrafish (36 months (~85% of lifespan)). (**C**) Zoomed-in images of the yellow boxes shown in panels (**A**) and (**B**); top image = young, bottom image = aged. (**D**) Cross-sectional myofiber area was quantified in H&E sections obtained from the caudal musculature of young and aged animals (*n* = 14). ** *p* ≤ 0.01.

**Figure 4:**
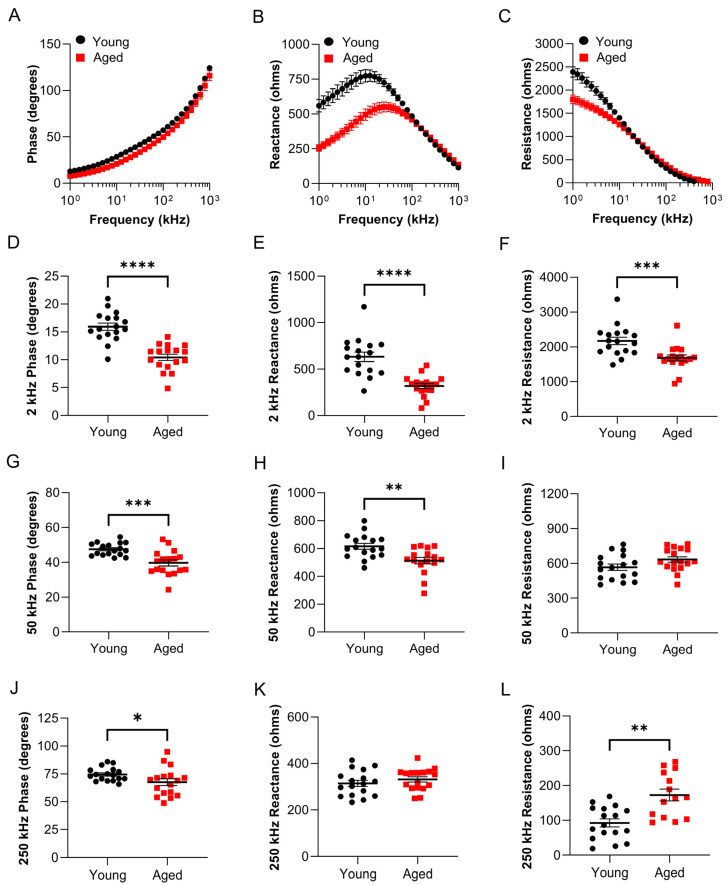
Surface electrical impedance myography detects age-related muscle changes in wildtype zebrafish. sEIM parameters (phase, reactance, and resistance) were assessed at a range of frequencies (1 kHz–1 MHz) in the epaxial caudal muscles of young (8 months, ~20% lifespan; *n* = 17) and aged zebrafish (36 months, ~85% lifespan; *n* = 17). Multifrequency graphs are shown for 3 impedance parameters: (**A**) phase, (**B**) reactance, and (**C**) resistance. Single frequency analyses at (**D**) 2 kHz phase (*p* < 0.000001, q = 0.000002), (**E**) 2 kHz reactance (*p* = 0.000004, q = 0.000006), (**F**) 2 kHz resistance (*p* = 0.000867, q = 0.000683), (**G**) 50 kHz phase (*p* = 0.000252, q = 0.000265), (**H**) 50 kHz reactance (*p* = 0.006081, q = 0.003192), (**I**) 50 kHz resistance, (**J**) 250 kHz phase (*p* = 0.044723, q = 0.020125), (**K**) 250 kHz reactance, (**L**) 250 kHz resistance (*p* = 0.001384, q = 0.000872). * *p* ≤ 0.05, ** *p* ≤ 0.01, *** *p* ≤ 0.001, **** *p* ≤ 0.0001.

Surface electrical impedance myography detects altered bioelectrical impedance in the caudal muscles of aged wildtype zebrafish. We classified animals that were 8–12 months of age (~20–30% of lifespan) as “young” and animals that were 36 months of age (~85% of lifespan) as “aged”, as described in the methods. Skeletal muscle architecture in young and aged zebrafish was quantified in H&E sections obtained from the caudal musculature. Young animals exhibit large polygonal-shaped muscle fibers, while aging animals exhibit morphological changes in skeletal muscle (Figure 3A–C), specifically, ~25% decreased myofiber size (Figure 3D; *p* = 0.003, *n* = 14). sEIM parameters of phase, reactance, and resistance across a range of 41 frequencies (1 kHz–1 MHz) were measured in the epaxial caudal muscles of young (8 months) and aged zebrafish (36 months) (Figure 4A–C; *n*= 17). In aged animals, there was an overall trend toward decreased phase at low frequencies (<10 kHz), decreased reactance at almost all frequencies, decreased resistance at frequencies <60 kHz, and increased resistance at high frequencies (250 kHz). In single frequency analyses, the most significant findings were at 2 kHz phase (*p* < 0.000001, q = 0.000002) and 2 kHz reactance (*p* = 0.000004, q = 0.000006), followed by 50 kHz phase (*p* = 0.000252, q = 0.000265) and 2 kHz resistance (*p* = 0.000867, q = 0.000683) (Figure 4D–L). Additional significant findings were 250 kHz resistance (*p* = 0.001384, q = 0.000872), 50 kHz reactance (*p* = 0.006081, q = 0.003192), and 250 kHz phase (*p* = 0.044723, q = 0.020125) (Figure 4D–L). We conducted impedance measurements in a second independent cohort of young and aged animals, which demonstrated similar findings (Figure S4A–L).

**Figure 5:**
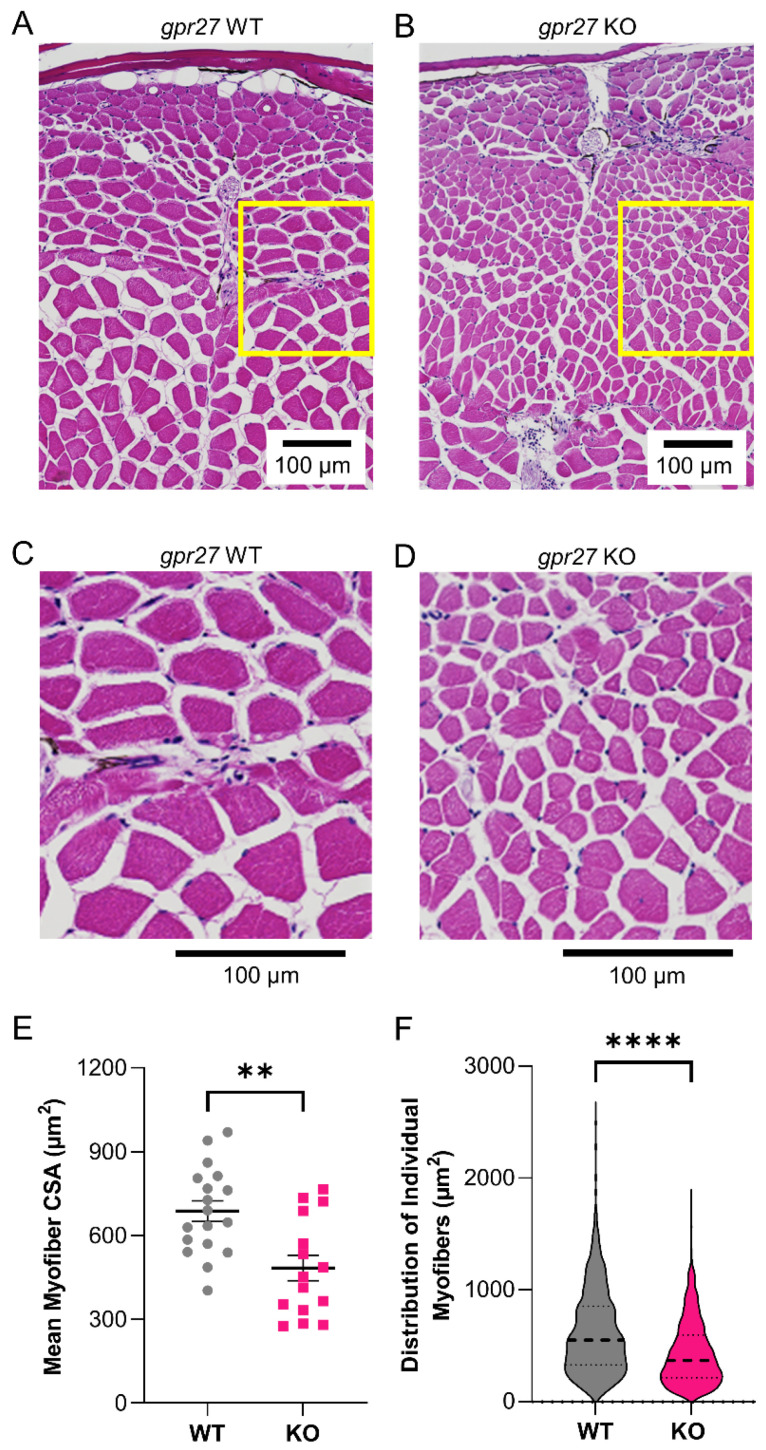
Aged *gpr27* KOs exhibit greater myofiber atrophy as compared to aged sibling controls. Representative H&E-stained images depicting epaxial caudal musculature in (**A**) aged WT (36 months; 85% lifespan) and (**B**) aged *gpr27* KO zebrafish (36 months; 85% lifespan). (**C**) Zoomed-in image of the yellow box shown in panel (**A**). (**D**) Zoomed-in image of the yellow box shown in panel (**B**). (**E**) Cross-sectional myofiber area and (**F**) distribution of the sizes of individual myofibers were quantified in H&E sections obtained from the caudal musculature of aged WT and aged KO animals (*n* = 15–18). ** *p* ≤ 0.01, **** *p* ≤ 0.0001.

Genetic deletion of *gpr27* exacerbates the atrophy of skeletal muscle in aged animals. We chose to assess this specific gene because it is part of an ongoing project aimed at assigning biological functions to understudied and druggable therapeutic targets, including orphan GPCRs [40]. In prior work, we detected defects in skeletal muscle function in *gpr27* KOs as compared to sibling WT controls. Skeletal muscle is responsible for the post-prandial disposal of ~80% of glucose [44,45]; *gpr27* KOs exhibit defects in insulin-mediated glucose uptake [40]. Moreover, in histological analyses of gross organ morphology, we found that aged *gpr27* KOs exhibited structural changes in skeletal muscle tissue as compared to aged WT siblings (Figure 5A–D). Aged WT zebrafish exhibited a decrease in myofiber size as compared to young WT zebrafish, as expected (Figure 5A, 36 months, versus Figure 3A, 8 months). However, aged *grp27* KOs (36 months) exhibited an exacerbated reduction in myofiber size as compared to aged WT siblings (36 months) (Figure 5A–D). This difference was significant, as demonstrated by the 30% decrease in cross-sectional myofiber area in aged *gpr27* KOs as compared to aged WT siblings (483.7 ± 176.6 versus 687.2 ± 156.1; *p* = 0.002, Figure 5E). In addition, there was a shift in the size distribution of the myofibers (Figure 5F, *p* < 0.0001).

**Figure 6:**
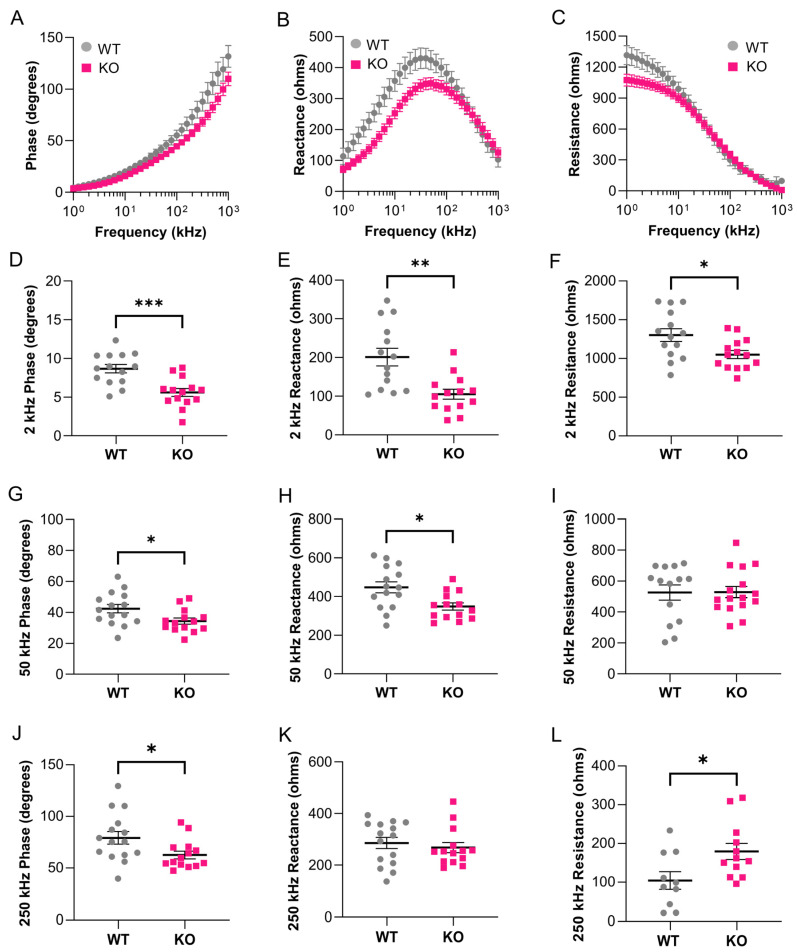
Genetic deletion of *gpr27* exacerbates age-related atrophy of caudal muscle. sEIM parameters were measured at frequencies of 1 kHz–1 MHz in the epaxial caudal muscles of aged sibling WTs (36 months, 85% lifespan, *n* = 14) and aged *gpr27* KOs (36 months, 85% lifespan, *n* = 14). Multifrequency graphs for (**A**) phase, (**B**) reactance, and (**C**) resistance (*n* = 14 per genotype). Single frequency analyses at (**D**) 2 kHz phase (*p* = 0.0006, q = 0.0012), (**E**) 2 kHz reactance (*p* = 0.0011, q = 0.0012), (**F**) 2 kHz resistance (*p* = 0.0273, q = 0.0082), (**G**) 50 kHz phase (*p* = 0.0229, q = 0.0082), (**H**) 50 kHz reactance (*p* = 0.0101, q = 0.0070), (**I**) 50 kHz resistance, (**J**) 250 kHz phase (*p* = 0.0258, q = 0.0082), (**K**) 250 kHz reactance, (**L**) 250 kHz resistance (*p* = 0.0249, q = 0.0082). * *p* ≤ 0.05, ** *p* ≤ 0.01, *** *p* ≤ 0.001.

sEIM measurements were acquired across a range of 41 frequencies (1 kHz–1 MHz) in the epaxial caudal muscles of aged *grp27* KO zebrafish (36 months) and compared to aged WT zebrafish (36 months; *n* = 14 per genotype). Note, for this study, WT sibling controls and *gpr27* KO animals were derived from heterozygous incrosses. There was not a statistically significant difference in weight (WT = 431 ± 106 mg and KO = 397 ± 75 mg) or standard length (WT = 2.96 ± 1.9 cm and KO = 2.95 ± 1.1 cm) between groups. In aged WT animals, we observed similar trends in phase, reactance, and resistance values across the frequency spectrum, as shown above in aged animals (Figure 6A–C versus Figure 4A–C). In aged *gpr27* KOs as compared to aged sibling WTs, there was an overall trend toward decreased phase at almost all frequencies, decreased reactance at frequencies <50 kHz, and decreased resistance at low frequencies <10 kHz. In single-frequency analyses (Figure 6D–L), the most significant findings were at 2 kHz phase (*p* = 0.0006, q = 0.0012) and 2 kHz reactance (*p* = 0.0011, q = 0.0012). Additional significant findings were 2 kHz resistance (*p* = 0.027, q = 0.0082), 50 kHz phase (*p* = 0.0229, q = 0.0082), 50 kHz reactance (*p* = 0.0101, q = 0.0070), 250 kHz phase (*p* = 0.0258, q = 0.143), and 250 kHz resistance (*p* = 0.0249, q = 0.0082).

**Figure 7:**
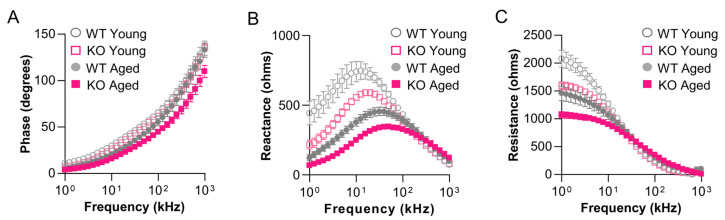
Surface electrical impedance myography detects skeletal muscle defects in young *gpr27* KOs. sEIM parameters were measured at frequencies of 1 kHz–1 MHz in the epaxial caudal muscles of young sibling WTs (12 months; 30% lifespan) and young *gpr27* KOs (12 months; 30% lifespan). Multifrequency graphs for (**A**) phase, (**B**) reactance, and (**C**) resistance in young animals (*n* = 12–14 per genotype). Note the data from aged WT and *gpr27* KO animals from Figure 6 (36 months; 85% lifespan) were also plotted for comparison (*n* = 14 per genotype).

Surface electrical impedance myography detects skeletal muscle defects in young *gpr27* KOs. Having shown that loss of *gpr27* causes exacerbated skeletal muscle defects in aged KO animals (36 months old; 85% lifespan) which were quantitatively detected by sEIM (Figure 5 and Figure 6), we next sought to determine if younger *gpr27* KO animals also exhibit defects that are detectable by sEIM. In this study, animals were assessed at 12 months (~30% lifespan). Across a range of frequencies (1 kHz–1 MHz), sEIM was measured in the epaxial caudal muscles of young *grp27* KO zebrafish (12 months) and compared to young sibling WT zebrafish (12 months). In *gpr27* KOs, there was an overall trend toward decreased phase at almost all frequencies, decreased reactance at frequencies <50 kHz, and decreased resistance at low frequencies <10 kHz (Figure 7; *n* = 12–14 per genotype). In single-frequency analyses (Supplementary Figure S5), the significant findings were at 2 kHz reactance (*p* = 0.0004, q = 0.0282), 2 kHz resistance (*p* = 0.0126, q = 0.0311), 50 kHz reactance (*p* = 0.0148, q = 0.0311), and 2 kHz phase (*p* = 0.0233, q = 0.0367). Thus, prior to the advanced atrophied muscle in *gpr27* KOs at 36 months of age, *gpr27* KOs begin to exhibit defects in skeletal muscle electrophysiology at 12 months of age, albeit in a subset of EIM parameters.

**Figure 8:**
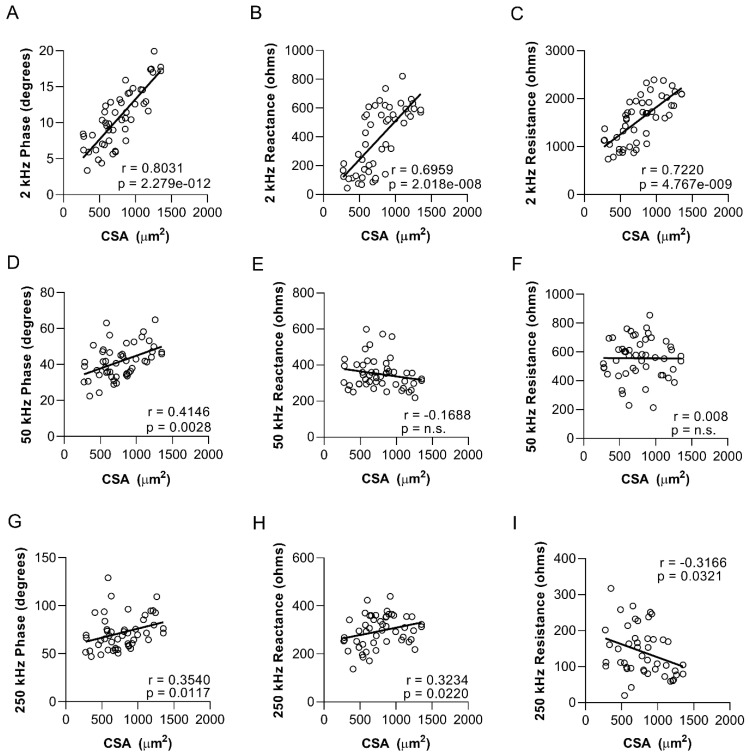
Myofiber size in zebrafish skeletal muscle strongly correlates with low-frequency phase, reactance, and resistance values. Correlation between cross-sectional myofiber area in the epaxial caudal musculature and sEIM values: (**A**) 2 kHz phase, (**B**) 2 kHz reactance, (**C**) 2 kHz resistance, (**D**) 50 kHz phase, (**E**) 50 kHz reactance, (**F**) 50 kHz resistance, (**G**) 250 kHz phase, (**H**) 250 kHz reactance, and (**I**) 250 kHz resistance (*n* = 50). Analyses included young (8 months; 20% lifespan) and aged (36 months; 85% lifespan) zebrafish data combined. Each panel displays Spearman r and *p* values.

Cross-sectional myofiber area correlates with surface electrical impedance myography measurements in zebrafish skeletal muscle. Electrical impedance is known to correlate with histological and morphometric features of muscle fibers in animals and humans [31,46,47,48]. We evaluated the correlation between sEIM values and cross-sectional myofiber area, as assessed by morphometric analysis on skeletal muscle tissue sections from zebrafish (*n* = 50). Strong associations were found between myofiber size and 2 kHz phase (r = 0.831; *p* = 2 × 10^−12^), 2 kHz reactance (r = 0.6959; *p* = 2 × 10^−8^), and 2 kHz resistance (r = 0.7220; *p* = 4 × 10^−9^) (Figure 8A–C). Nominally significant findings were also observed at other frequencies (Figure 8G–I).

**Figure 9:**
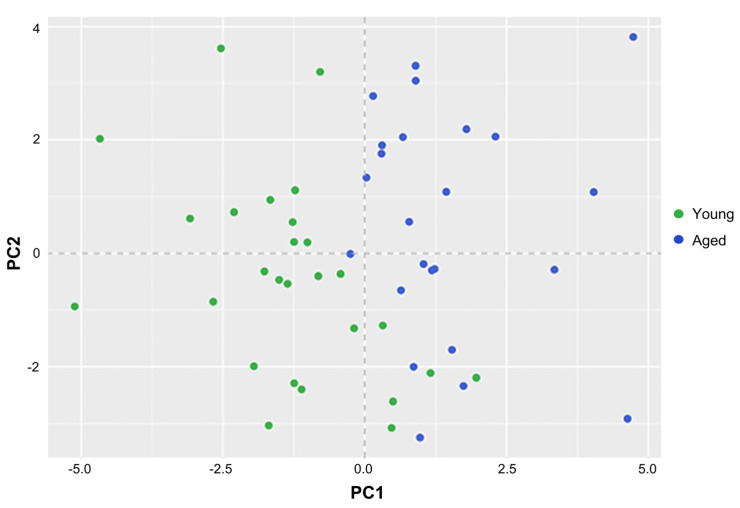
Principal component analysis of EIM measurements in young and aged zebrafish. Nine EIM parameters were used in this analysis (phase: 2, 50, and 250 kHz; reactance: 2, 50, and 250 kHz; and resistance: 2, 50, and 250 kHz). Data from 51 animals was used (*n* = 27 young, i.e., 8 months, and *n* = 24 aged, i.e., 36 months). The first two components of the PCA explained 86.2% of the variance between the young and aged zebrafish. The EIM parameters of phase at 2 kHz phase, resistance at 2 kHz resistance, and reactance at 2 kHz, 50 kHz, and 250 kHz contributed to >80% of PC 1. Resistance at 50 kHz and 250 kHz and phase at 50 kHz and 250 kHz contributed to >80% of PC 2.

Principal component analysis of EIM measurements in young and aged zebrafish. To identify the set of EIM parameters that accounts for the greatest variance in the dataset, we used principal component analysis (PCA) to reduce the dimensionality of the data. Nine EIM parameters were used in this analysis (phase: 2, 50, and 250 kHz; reactance: 2, 50, and 250 kHz; and resistance: 2, 50, and 250 kHz). We pooled two cohorts, each containing young and aged animals (Figure 4 and Supplementary Figure S4), excluding animals that had missing data values; EIM data from 51 animals were used (*n* = 27 young and *n* = 24 aged). The first two components of the PCA explained 86.2% of the variance between the young and aged zebrafish (Figure 9). The EIM parameters of phase at 2 kHz phase, resistance at 2 kHz resistance, and reactance at 2 kHz, 50 kHz, and 250 kHz contributed to >80% of principal component (PC) 1. Resistance at 50 kHz and 250 kHz and phase at 50 kHz and 250 kHz contributed to >80% of PC 2.

Prediction modeling. We next sought to determine if age could be predicted using a subset of EIM variables. We considered this a pilot analysis because of the small number of animals used. Typically, prediction modeling utilizes large numbers of subjects, and here, we used only two ages (8 and 36 months) as opposed to assessing multiple ages throughout the lifespan. In this pilot analysis, we independently analyzed cohort 1 (Figure 4) and cohort 2 (Supplementary Figure S4). We attempted to derive a statistical model that would focus on the most relevant EIM features. The EIM parameters of phase at 2 kHz and 50 kHz and reactance at 2 kHz were selected as predictors of young versus aged zebrafish using a LASSO logistic regression model derived from splitting 80% of the cohort 1 results (Figure 4) into a training dataset and 20% into a testing dataset. The model was tuned to an alpha = 1 (since LASSO was used), and lambda associated with the lowest CV value (lambda = 0.02). This prediction model was then tested on the testing dataset with an AUC of 1. This result is likely optimistic given that only six animals were represented in the testing dataset. Therefore, the model was used to segregate young versus aged animals in cohort 2 (Supplementary Figure S4) with an AUC of 0.75. Thus, this pilot analysis demonstrates that selected EIM parameters can accurately classify a zebrafish as “aged”.

## 4. Discussion

Surface electrical impedance myography has been successfully employed in several mammalian species. Our study brings the zebrafish model to this growing body of work, which aims to leverage impedance measurements as a biomarker of disease progression and treatment outcome for skeletal muscle disorders. Due to the experimentally expedient nature of the zebrafish model, our new platform will provide a new approach for go/no-go decisions for small molecules and therapeutic targets that affect skeletal muscle health.

Here, we developed a surface electrical impedance myography methodology in adult zebrafish to noninvasively and quantitatively measure skeletal muscle health; to our knowledge, this has not been previously reported in zebrafish. Using the sEIM platform described here, skeletal muscle health was assessed in young animals, aged animals, and *gpr27* KO animals. This study builds upon our prior work reporting the first zebrafish study that used needle electrical impedance myography to assess skeletal muscle properties in adult zebrafish, which were albeit euthanized [42]. In that study, needle EIM was used; in needle EIM, the electrode is in direct contact with the tissue of interest, as it is physically inserted into the muscle tissue. However, due to the small size of zebrafish, needle EIM is a terminal procedure. This was an important first study that began to establish the “true” skeletal muscle impedance properties in zebrafish. However, for electrical impedance myography to be a valuable tool for investigating age-related muscle deficits in zebrafish and for assessing the therapeutic potential of genetic and pharmaceutical interventions in this model organism, it must be performed noninvasively in live zebrafish. Thus, in the present study, we developed a method to conduct surface EIM on zebrafish (i.e., placing the electrode on the descaled skin overlying the muscle of interest rather than inserting it into the muscle tissue).

To date, most studies applying EIM for muscle assessment in both humans and in animals have utilized surface methods [49]. Surface methods have clear benefits. First, they are non-invasive, which means that they can be applied comfortably in humans and animals. For example, in humans, surface EIM can be performed on sleeping newborns and older children [50,51,52]. Similarly, surface EIM has been performed on awake dogs [53,54,55]. While other animals, including mice [56], rats [32], and, here, zebrafish, must be studied under anesthesia to keep them from moving while the fur or scales are removed and data collected, sEIM still remains relatively unintrusive. Its noninvasive nature speaks to a second critical feature of surface EIM—namely, the ability to measure animals repeatedly over time to quantitatively assess disease progression or the impact of a therapy without altering the muscle condition in the process. This ability has been used to great advantage in the use of sEIM for assessing conditions such as ALS and muscular dystrophy in both human and animal models [52,53,54]. Tracking change over time and the impact of a treatment are two of the perhaps most valuable aspects of sEIM technology. Achieving a similar capability in zebrafish means that drug therapies can be tested effectively in disease states.

The data here show that surface EIM techniques can effectively discriminate between young healthy muscle and aged sarcopenic muscle (Figure 3 and Figure 4, and Supplementary Figure S4), as well as the subtly atrophied muscle in the *gpr27* KO animals. In *gpr27* Kos, the defects in skeletal muscle progressively develop, first beginning to appear in young animals (~30% lifespan), as evidenced by changes in a subset of sEIM parameters (Supplementary Figure S5). However, a more pronounced atrophy is present at advanced age (~85% lifespan) in *gpr27* KOs as compared to aged sibling WTs (Figure 5 and Figure 6). These findings suggest that *gpr27* may play a role in skeletal muscle health and the development of sarcopenia. The molecular mechanism by which *gpr27* affects myofiber size is not known. However, it is possible that the metabolic actions of this GPCR [40] also affect skeletal muscle growth, the maintenance of cell size, and/or myofiber survival; future work is this area is warranted.

The studies presented here also show how surface EIM values correlate with cell size across the animals, making it potentially possible to utilize sEIM in zebrafish as a tool to noninvasively assess myofiber atrophy, a valuable measure for muscle and gerontology research (Figure 8). Coupled with earlier data showing a strong relationship between needle EIM values and swimming behaviors [42], EIM appears to offer an objective rapid measurement tool for future use in adult zebrafish. Applications of the technology could include tracking zebrafish muscle alterations more granularly as they age through the lifespan, assessing the impact of drug therapies to slow aging-related changes or specifically to treat or correct genetic or other acquired disorders. Indeed, our next goal will be to study the use of EIM in animals exposed to therapy that induces myofiber atrophy (dexamethasone added to the water) and recovery.

Surface EIM measurements have clear advantages. Nevertheless, there are also some associated challenges to the technique. First, in general, obtaining surface measurements can be challenging since the skin needs to be relatively moist, hair/scale free, and smooth. Humans are, fortunately, ideal to study in this regard because there is relatively little body hair. Mice are perhaps the most challenging since they are very small, require minute electrodes, and they have dense fur that needs to be removed with a depilatory agent. Moreover, depilatory-agent-mediated hair removal treatments in rodents causes irritation artifacts and a scab that can take 1–2 weeks to fully recover. The application of sEIM here in zebrafish, regardless of category (young, old, and genetically modified) was simpler and faster compared to analogous surface approaches in mice and rats, given the ease with which the scales can be removed (i.e., simply using tweezers to pluck 8–12 scales in zebrafish). In contrast to the irritation caused by hair removal in rodents, scale removal in zebrafish is relatively non-injurious, and the scales can grow back quickly in a matter of only days. Another disadvantage to surface EIM measurements is that the skin and subcutaneous tissues overlying a muscle will impact the acquired data to some extent [55]. Since muscle is far more conductive than fat or skin, the electrical current preferentially travels through this tissue, providing mainly a muscle measure. However, with increasing subcutaneous fat, less and less current can effectively reach the muscle, and the impedance data become increasingly contaminated and influenced by the fat [55,57]. Notably, in zebrafish, as compared to mice, the electrode is in closer proximity to the tissue of interest, i.e., the skeletal muscle (Figure 1); thus, the impedance data are less impacted by the resistive and conductive properties of tissues between the electrode and the skeletal muscle (i.e., stratum corneum, epidermis, hypodermis, etc., in mice). The layers of tissue that overlay zebrafish skeletal muscle, i.e., the epidermis, scales, and dermis, are ~25 µm thick, while in mice, the tissue layers that overlay the skeletal muscle, i.e., epidermis, dermis, hypodermis, and connective tissue, are ~300 µm thick. Moreover, in zebrafish, the epidermis encases each scale. Therefore, removing a region of scales also removes the epidermis in that region, placing the electrode on the thin dermis that overlays the skeletal muscle.

Indeed, we have shown that with the removal of the scales, high-quality impedance data can be obtained with relatively little effort and with minimal contact artifacts. This can be appreciated most clearly in Figure 4, which shows robust reactance and resistance curves with virtually no low-frequency artifacts (generally very high values at very low frequencies that eventually normalize at about 20–40 kHz). In fact, the data from the very lowest frequencies obtained are impressively artifact-free. The strong correlation to needle data across much of the frequency range supports this and follows what was previously reported in mice ex vivo data [58]. Indeed, the needle measurement taken immediately after a surface measurement was taken showed how surface EIM correlated with homologous needle EIM parameters, especially at lower frequencies (Figure 2).

Still, there were noticeable differences between the surface and needle data. There are several likely reasons for this. First, the needles are injurious to the tissue and may produce localized edema and bleeding which will clearly alter measured impedance. The electrodes themselves are also different sizes and shapes, and thus the absolute values obtained are going to be considerably different on that basis alone. This likely explains why the resistance and reactance (and to a lesser extent, the phase values) are generally lower in the needle measurements as compared to the surface. Namely, localized tissue injury and a larger surface area in the needle probes will result in different data. Another factor, which is more complex, likely relates to more complicated features of impedance measurements, including regions of positive and negative sensitivity in which impedance features in certain locations or at certain frequencies may behave in unexpected or even opposite ways [59]. The theoretical details of this go far beyond this article but could account for some of the negative correlations observed at higher frequency and underscores the limitations of such comparative analyses.

There are some limitations to this study. First, we studied only animals whose most obvious pathology is myofiber atrophy, i.e., aged animals. However, sEIM is sensitive to other compositional and structural elements, including fat deposition and inflammation [60,61], which would be expected to alter the surface sEIM data in predictable ways in zebrafish. In addition, sEIM is sensitive to other models of muscle dystrophy, including ALS [32,34,35,36]. Therefore, future studies are required in zebrafish models of obesity and inflammation, in addition to genetic models of muscular dystrophies. Second, the procedure in zebrafish remains relatively new, and there are likely to be additional refinements made to the electrode array and measurement technique as we attempt to further streamline our measurement approaches for high-throughput chemical screening. For example, although the protocol is fast, an additional modification that could further increase throughput is the development of a multi-pronged electrode which could simultaneously acquire data on more than one zebrafish at a time. Third, we have not yet attempted to collect longitudinal data in the same animal over an extended period of time—beyond testing two time points spanning 14 days in the same animal (and the same region of the musculature). Thus, we do not know the potential longer-term issues with repeated scale removal in the same exact region on our animals or if multiple adjacent regions will need to be tested to avoid this potential issue. Fourth, we have not attempted to assess more detailed features of muscle histology, including myofiber type (white versus red) or connective tissue deposition. Fifth, our statistical prediction model of age was a pilot analysis (Figure 9). In future studies, we will use large numbers of subjects in addition to multiple ages through the lifespan of zebrafish.

In conclusion, we have developed a novel technique in adult zebrafish to allow for the rapid assessment of muscle health. The platform is highly sensitive to myofiber size and condition, and given its relative ease of application and that it is non-invasive, it could be applied repeatedly in animals undergoing longitudinal evaluation for disease progression or therapeutic study. Our future plans include such longitudinal studies, as well as further advances in data analytics associated with these measures. Indeed, recent work has pointed to the potential of utilizing machine learning methods to assist in the analysis of these complex multifrequency and multi-parametric data sets [62]. Applying these analytical methods to zebrafish data may help in extracting a richer set of information that will further our efforts in applying this technology to a large set of neuromuscular disorders.

## 5. Impact

By establishing surface electrical impedance myography methodology (a non-invasive and high-content tool) in the zebrafish model (an experimental tractability organism), we created a new and practical platform to enable go/no-go decisions for small molecules and therapeutic targets that affect skeletal muscle health. Importantly, as compared to murine and larger mammalian models, the establishment of non-invasive surface electrical impedance myography in zebrafish will be a springboard to many small molecule chemical screens focused on therapeutic discovery for sarcopenia, for which there is currently no treatment.

## Figures and Tables

**Table 1:** Intra-observer reproducibility of surface electrical impedance myography in zebrafish.

Impedance Parameter ^a^			
Frequency (kHz)	Phase	Reactance	Resistance
2	1.47 ± 0.39%	1.92 ± 0.51%	1.32 ± 0.35%
50	1.39 ± 0.37%	1.05 ± 0.28%	2.87 ± 0.76%
250	1.22 ± 0.32%	2.35 ± 0.62%	1.99 ± 0.53%

kHz: kilohertz. ^a^ Reproducibility was assessed by calculating the mean percent difference between serial measurements acquired without moving the electrode.

## Data Availability

Data is contained within the article or Supplementary Material. The data reported in this paper is also available from the corresponding authors upon request.

## Supplementary Materials

The following supporting information can be downloaded at: https://www.mdpi.com/article/10.3390/biomedicines11071938/s1, Figure S1: Micromanipulator-assisted electrode approach and electrode placement on caudal musculature of a zebrafish. Figure S2: Surface electrical impedance myography measurements in zebrafish are reproducible between observers. Figure S3: Surface electrical impedance myography conducted on different days exhibits temporal reproducibility. Figure S4: Surface electrical impedance myography detects age-related muscle changes in zebrafish. Figure S5: Surface electrical impedance myography detects skeletal muscle defects in young *gpr27* Kos. Table S1. Inter-observer reproducibility of surface EIM in zebrafish.
