# Supplementary material for: Surface Electrical Impedance Myography Detects Skeletal Muscle Atrophy in Aged Wildtype Zebrafish and Aged gpr27 Knockout Zebrafish
Source: Biomedicines. 2023 Jul 7;11(7):1938. doi: 10.3390/biomedicines11071938 (PMC10377526; doi:10.3390/biomedicines11071938)
Supplement: Supplementary file 1 [file biomedicines-11-01938-s001.zip › biomedicines-2414434-supplementary.pdf]

SUPPLEMENTARY MATERIAL

Surface Electrical Impedance Myography Detects Skeletal Muscle Atrophy in Aged Wildtype Zebrafish and Aged *gpr27* Knockout Zebrafish

Seward B. Rutkove[1,2,‡], Zsu-Zsu Chen[3,4], Sarbesh Pandeya[1], Santiago Callegari[4], Tyler Mourey[5], Janice A. Nagy[1], and Anjali K. Nath[2,4,6,‡]

[1]Department of Neurology, Beth Israel Deaconess Medical Center, Boston, MA, 02215, USA.
[2]Harvard Medical School, Boston, MA 02215, USA.
[3]Department of Endocrinology, Beth Israel Deaconess Medical Center, Boston, MA 02215, USA.
[4]Department of Cardiology, Beth Israel Deaconess Medical Center, Boston, MA 02215, USA.
[5]Zebrafish Core Facility, Beth Israel Deaconess Medical Center, Boston, MA 02215, USA.
[6]Broad Institute, Cambridge, MA 02142, USA.
[‡]To whom correspondence should be addressed: Anjali K. Nath (anath1@bidmc.harvard.edu) and Seward B. Rutkove (srutkove@bidmc.harvard.edu)

SUPPLEMENTARY FIGURES

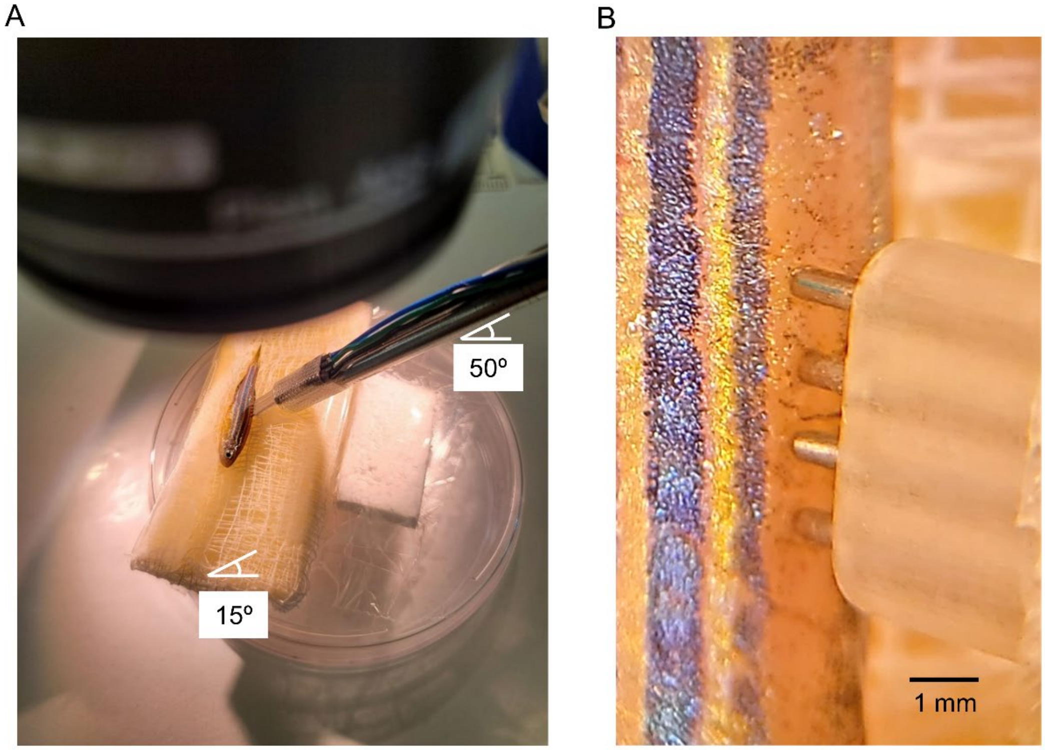

Supplementary Figure S1. Micromanipulator-assisted electrode approach and electrode placement on caudal musculature of a zebrafish. A) An anesthetized zebrafish was placed on a 15º angle wedge platform. The surface electrode was attached to a micromanipulator which was set at a 50º angle to the horizon. **B)** Zoomed-in image demonstrating the placement of the 4-pin surface electrode on the descaled epaxial caudal musculature. The electrode was positioned on the anterior-posterior axis and below the dorsal fin.

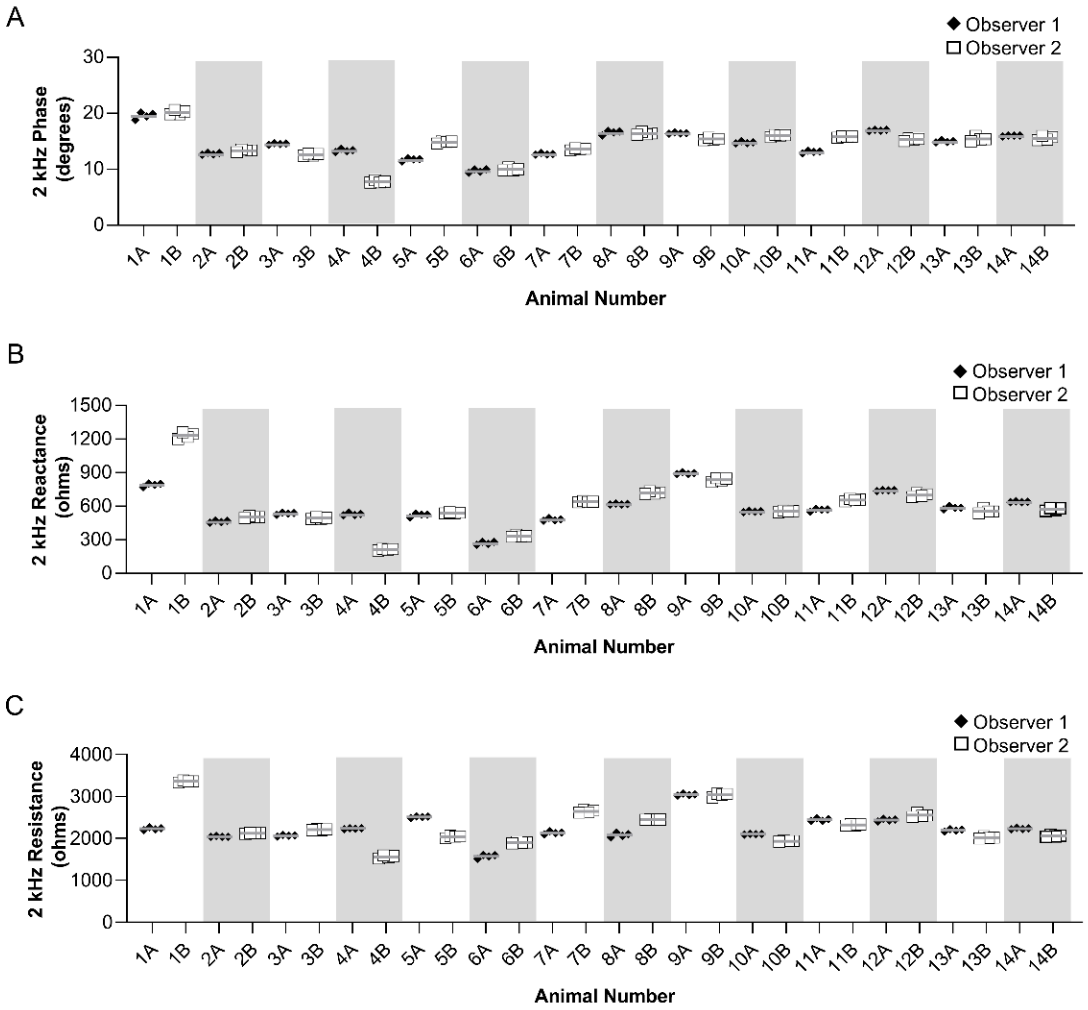

Supplementary Figure S2. Surface electrical impedance myography measurements in zebrafish are reproducible between observers. Values for impedance parameters: **A)** phase angle **B)** reactance, and **C)** resistance. Observer 1 (black diamonds) and Observer 2 (white squares) each independently acquired 4 serial readings on the same animal (the gray line denotes the mean value acquired by each Observer on each animal, n=14 young zebrafish, 8 months of age). The surface electrode was positioned on the animal by Observer 1 (black diamonds) and 4 serial measurements were captured. Next, the electrode was removed by Observer 1. Observer 2 (white squares) positioned the electrode and captured 4 additional serial measurements in the animal. Inter-observer reproducibility was further evaluated by calculating the mean percent difference between these repeated measures in the same animal conducted by two different observers; see Supplementary Table S1 below.

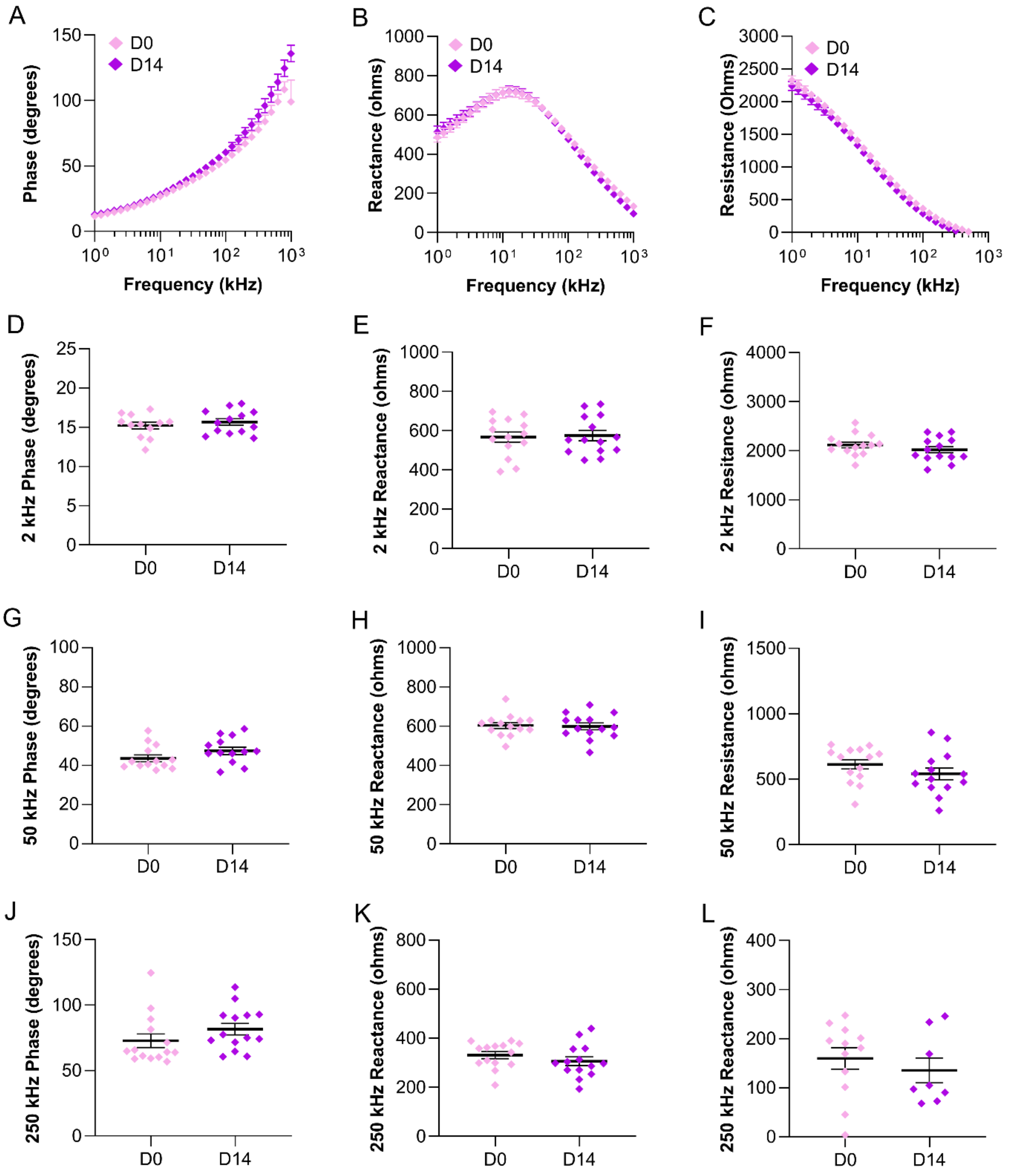

Supplementary Figure S3. Surface electrical impedance myography conducted on different days exhibits temporal reproducibility. To determine the temporal reproducibility of sEIM measurements in zebrafish skeletal muscle, data were acquired on different days (D0 and D14) in the same cohort of young animals (n=14). Multifrequency graphs (1 kHz – 1 MHz) for **A)** phase angle, **B)** reactance, and **C)** resistance. Single frequency analyses at **D)** 2 kHz phase, **E)** 2 kHz reactance, **F)** 2 kHz resistance, **G)** 50 kHz phase, **H)** 50 kHz reactance, **I)** 50 kHz resistance, **J)** 250 kHz phase, **K)** 250 kHz reactance, and **L)** 250 kHz resistance.

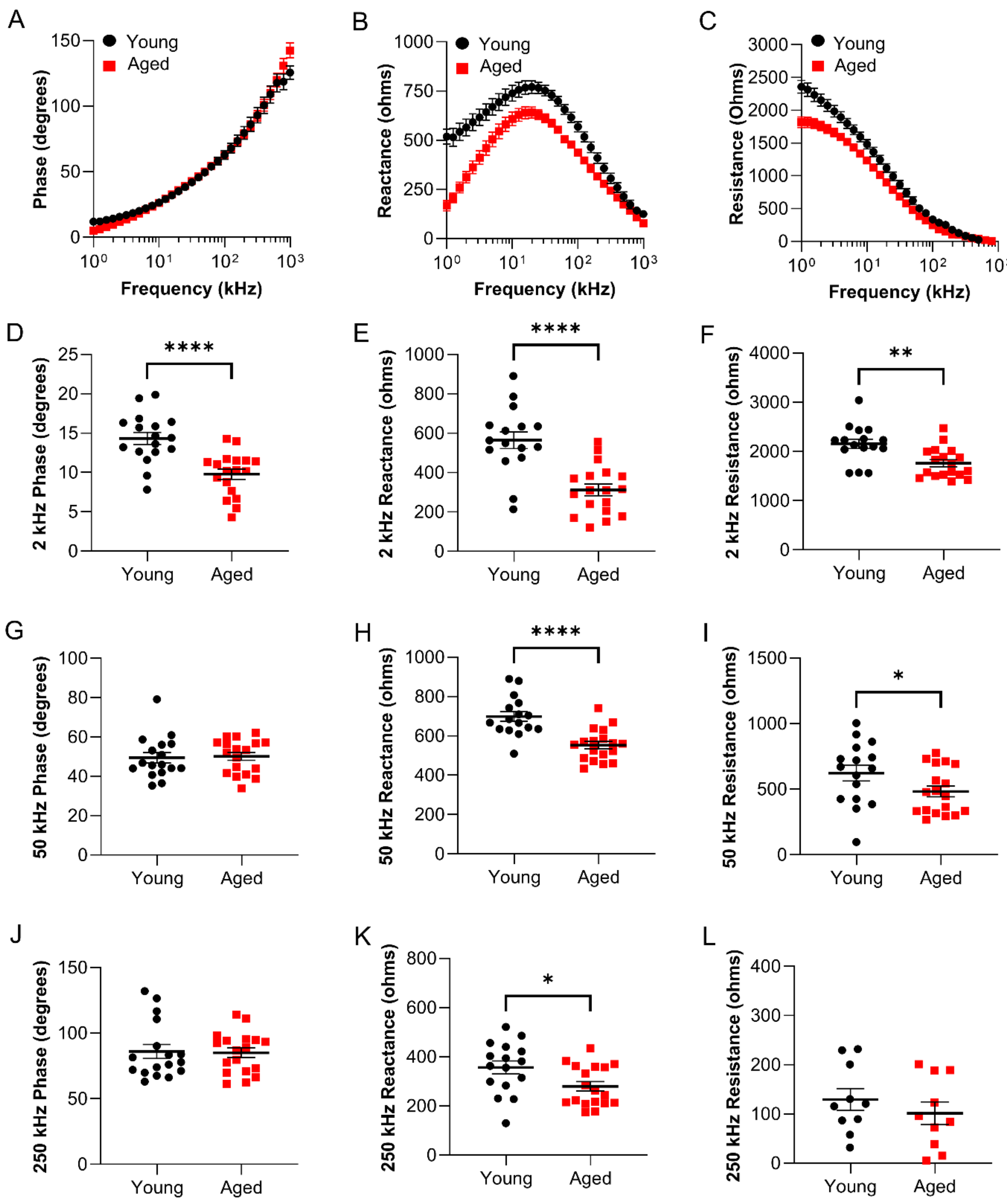

Supplementary Figure S4. Surface electrical impedance myography detects age-related muscle changes in zebrafish. In a replication cohort, sEIM parameters across a range of frequencies (1 kHz – 1 MHz) were measured in the epaxial caudal muscles of young (8 months) and aged zebrafish (36 months). Multifrequency graphs for **A)** phase, **B)** reactance, and **C)** resistance (n=17-20 animals per age group). Single frequency analyses at **D)** 2 kHz phase (p=0.000047, q=0. 0.000091), **E)** 2 kHz reactance (p= 0.000044, q= 0.000091), **F)** 2 kHz resistance (p= 0.001553, q= 0.001631), **G)** 50 kHz phase, **H)** 50 kHz reactance (p=0.000065, q= 0.000091), **I)** 50 kHz resistance (p= 0.045282, q= 0.031697), **J)** 250 kHz phase, **K)** 250 kHz reactance (p= 0.015062, q=0.007908), **L)** 250 kHz resistance. * p ≤ 0.05, ** p ≤ 0.01, **** p ≤ 0.0001.

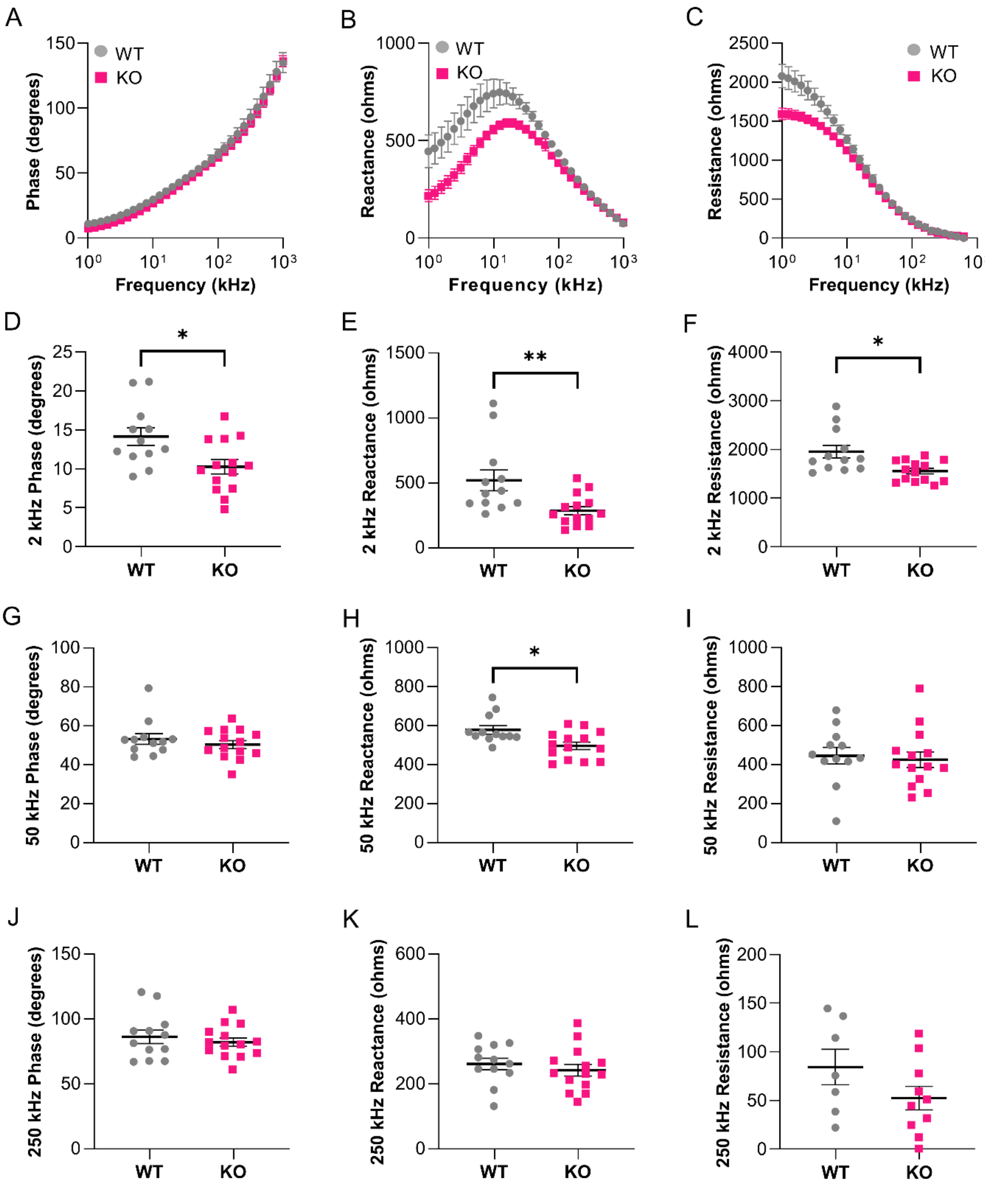

Supplementary Figure S5. Surface electrical impedance myography detects skeletal muscle defects in young *gpr27* KOs. sEIM parameters across a range of frequencies (1 kHz – 1 MHz) were measured in the epaxial caudal muscles of young *gpr27* KOs (12 months) and sibling WTs (12 months). Multifrequency graphs for **A)** phase, **B)** reactance, and **C)** resistance (n=12-14). Single frequency analyses at **D)** 2 kHz phase (p=0.0233, q=0.0367), **E)** 2 kHz reactance (p=0.0004, q=0.0282), **F)** 2 kHz resistance (p=0.0126, q=0.0311), **G)** 50 kHz phase, **H)** 50 kHz reactance (p=0.0148, q=0.0311), **I)** 50 kHz resistance, **J)** 250 kHz phase, **K)** 250 kHz reactance, **L)** 250 kHz resistance. * p ≤ 0.05, ** p ≤ 0.01.

Supplementary Table S1. Inter-observer reproducibility of surface EIM in zebrafish.

Frequency (kHz)	Impedance Parameter[a]		
	Phase	Reactance	Resistance
2	6.76±3.57%	9.80±5.94%	8.39±3.22%
50	6.71±3.50%	12.29±4.20%	12.65±10.75%
250	4.35±5.52%	21.9±7.27%	17.56±4.88%

kHz: kilohertz

[a]Reproducibility was assessed by calculating the median percent difference between Observer 1 and Observer 2, i.e. the electrode was retracted and repositioned between Observers. See Supplementary Figure 2.
